# From Successful Ageing to Ageing Well: A Narrative Review

**DOI:** 10.1093/geront/gnae109

**Published:** 2024-08-14

**Authors:** Chloe Waddell, George Van Doorn, Garry Power, Dixie Statham

**Affiliations:** Institute of Health and Wellbeing, Federation University Australia, Ballarat, Victoria, Australia; Health Innovation and Transformation Centre, Federation University, Ballarat, Victoria, Australia; Institute of Health and Wellbeing, Federation University Australia, Ballarat, Victoria, Australia; Health Innovation and Transformation Centre, Federation University, Ballarat, Victoria, Australia; Institute of Health and Wellbeing, Federation University Australia, Ballarat, Victoria, Australia; Health Innovation and Transformation Centre, Federation University, Ballarat, Victoria, Australia; Institute of Health and Wellbeing, Federation University Australia, Ballarat, Victoria, Australia; Manna Institute, University of New England, Armidale, New South Wales, Australia

**Keywords:** Ageing well, Australia, Narrative review, Successful ageing

## Abstract

Since the term “successful ageing” was coined, diverse models and theories conceptualizing what it means to age successfully have been proposed. The current article outlines evidence suggesting that the use of “success” in conjunction with “ageing” is contentious, and thus, “ageing well” is recommended as an alternative term. This article also highlights the lack of consistency in approaches to successful ageing and argues for a more inclusive conceptualization of ageing well. To achieve this, the current article summarizes the fundamental characteristics of several popular models of ageing successfully, demonstrating the unique contributions of each and highlighting recurring themes. The most common themes in existing models of successful ageing include the importance of engaging in social relationships, good cognitive and physical functioning, the avoidance of disease and disability, and resilience. Although commonalities exist, a consensus regarding an accepted definition of successful ageing is yet to be reached. To illustrate the need for consensus, policy approaches to support ageing populations by several governments are compared, highlighting the need for researchers to provide clearer guidance to policy-makers. In addition, not all existing models are sensitive to the diversity of the ageing population, further emphasizing the need to reconsider what it means to age well. The development of a consensus understanding of ageing well will improve the ability of researchers, as well as policy-makers and client-facing workers, to effectively target areas that contribute to, and improve, individuals’ ability to age well.

Our current understanding of what it means to age well is limited. Rowe and Kahn (1987) introduced the term “successful ageing,” defining it as an absence of disease and disability, high levels of physical and cognitive functioning, and engagement in interpersonal relationships and productive activities. Since then, researchers have attempted to identify and measure a range of factors thought to contribute to the quality of life in older adults. However, work in this space has been impeded by a lack of consensus regarding a definition, with terms such as healthy and active ageing used interchangeably with successful ageing. This lack of agreement has implications for researchers’ ability to operationalize, and thus measure, successful ageing. In this article, we use a narrative review to identify and synthesize key literature in the area (Sukhera, 2022a). In this review, and consistent with nonsystematic, narrative reviews, we present an overview of the current state of knowledge relating to successful ageing and ageing well, identify limitations, and highlight the implications for future thinking in this area. To provide historical context and demonstrate the progression of theory development, consistent with Sukhera’s (2022b) recommendation, the key theories are presented chronologically. On the weight of the evidence, we argue that current conceptualizations of successful ageing are limited and propose that future work adopts the more inclusive term “ageing well” as an alternative to “successful ageing.”

## What Are Successful Ageing and Ageing Well?

It is difficult to define “successful ageing” as a consensus has yet to be reached. For example, a meta-analysis of 28 peer-reviewed studies found 29 definitions of “successful ageing” (Depp & Jeste, 2006). Most definitions (26 of the 29) referred to physical health or disability, whereas cognitive ability was considered an important aspect of successful ageing in 13 publications. Depp and Jeste (2006) demonstrated that demographic variables (e.g., gender, income) were not associated with the ability to age successfully. However, as acknowledged by the authors, this finding may have been a product of (a) sampling bias or (b) the use of composite measures of successful ageing that incorporate several aspects of functioning, some of which could potentially be influenced by demographics.

The terms “ageing well” and “successful ageing” are often used interchangeably (Balderas-Cejudo et al., 2020; Quigley et al., 2022). In fact, several terms including, but not limited to, ageing “actively” and “healthily” are used in literature and policy, and despite these labels being analogous to one another, a review by Fernández-Ballesteros et al. (2013) suggested that these terms encompass some differences in meaning. The lack of clarity as to whether ageing well is distinct from successful ageing, or whether it can be used interchangeably with successful ageing (which, as alluded to above, is not clearly defined), creates problems in research and policy/practice spaces. Later, we present significant theories and perspectives on successful ageing and ageing well that have informed definitions and conceptualizations.

### Model of Successful Ageing

Rowe and Kahn (1987) coined the term “successful ageing” to highlight the difference between typical and successful ageing. To age successfully, according to Rowe and Khan’s (1997) model, one must be in good health (i.e., lack disease or disability) and be physically and socially active, cognitively adept, and contributing to society. This model of successful ageing was praised by Whitley et al. (2016) as being an appropriate and objective measure of successful ageing, but they suggested successful ageing should be considered a spectrum, rather than a dichotomy. A spectrum would be more representative of ageing experiences and would allow age-related decline and compensation strategies to coexist.

### Model of Psychological Wellbeing

Ryff (1989) criticized previous definitions of successful ageing for focusing on perceived deficiencies (e.g., illness) and, consequently, proposed a six-domain model of psychological well-being that she applied to successful ageing. These domains include (1) self-acceptance (e.g., accepting one’s positive and negative traits), (2) having satisfying interpersonal relationships involving trust, (3) autonomy such as being independent and able to self-regulate behavior, (4) environmental mastery (e.g., feeling competent about engaging in activities), (5) purpose in life (e.g., having goals), and (6) personal growth (e.g., having a desire to improve).

In contrast to other models of successful ageing (e.g., Rowe & Kahn’s [1997] model), Ryff’s (1989) conceptualization of successful ageing does not explicitly incorporate disease, disability, or physical/cognitive functioning. Although the domain of “autonomy” (i.e., being independent) may influence (or be influenced by) disease, disability, or functioning—as there are plausible relationships between one’s ability to be independent and their physical and cognitive health—Ryff (1989) focused primarily on psychological well-being.

### Model of Selective Optimisation With Compensation

Baltes and Baltes’ (1990) Selective Optimisation With Compensation (SOC) model outlines an adaptation strategy that can be applied throughout the lifespan but is of relevance to “older adults” given common age-related changes, such as reduced physical functioning, and diminished views of the self that stem from negative stereotypes associated with ageing. Selection refers to focusing on a reduced number of tasks based on, for example, the individual’s interests, priorities, and abilities, and adjusting some expectations in response to age-related changes (Baltes & Baltes, 1990). Optimization refers to practicing and engaging in behaviors that enhance the quality of life, whereas compensation refers to increasing effort or using other internal (e.g., mnemonics) and external (e.g., hearing aid) mechanisms to maintain functioning despite some reduction in ability (Freund, 2008; Freund & Baltes, 2000). Together, these three aspects form a model of adaptation to help achieve successful ageing, despite constraints associated with ageing. Implementation of SOC strategies can lead to positive outcomes for older adults including, but not limited to, increased happiness (Teshale & Lachman, 2016), positive emotions, low levels of agitation, contentment with ageing, and reduced loneliness (Freund & Baltes, 1998).

### Lifespan Model of Successful Ageing

Similar to Baltes and Baltes’ (1990) SOC model, Schulz and Heckhausen’s (1996) model focuses on adaptation to mitigate age-related losses. In this context, successful ageing is associated with nurturing and promoting “primary control” across one’s lifespan to facilitate personal growth, whereas mitigating challenges such as cognitive decline. Primary control is associated with outward processes (e.g., behaviors) designed to influence one’s immediate environment such that it better fits the individual’s wants and needs (Heckhausen & Schulz, 1995). The model, in part, implies that individuals who maintain high levels of primary control across the lifespan are successful agers.

Although this implies a rigid definition of success, Schulz and Heckhausen (1996) acknowledge that success must be considered relative to individual circumstances and resources. As such, and in contrast to Rowe and Kahn’s (1997) view that physical functioning is essential for successful ageing, Schulz and Heckhausen’s (1996) model posits that someone born with physical limitations, for example, can still achieve success. In this case, using assistive aids (e.g., wheelchairs) can be a way of achieving primary control that provides an individual with opportunities to achieve goals. This model, therefore, provides more inclusive criteria for successful ageing than do some other models (e.g., Rowe & Kahn, 1987, 1997).

### Successful Ageing Scale

Reker’s (2009) conceptualization of successful ageing is an amalgamation of the four theories discussed earlier. Reker’s work highlights that, although differing perspectives regarding successful ageing exist, commonalities are present across theories and definitions (see Table 1). Social engagement, for example, is common to many of the outlined models and, although it is not explicitly mentioned in Baltes and Baltes’ (1990) model, social engagement may be relevant in particular contexts (e.g., social engagement could constitute an adaptation strategy). Reker’s Successful Ageing Scale categorizes key parts of the four theories within three factors: (1) “healthy lifestyle” (e.g., good physical and mental health), (2) “life engagement” (e.g., achieving goals, social engagement), and (3) “adaptive coping” (e.g., mental toughness, compensation). Adaptive coping refers to cognitive and behavioral strategies used to deal with stressors and reduce adverse stress-related effects (Mah et al., 2020).

**Table 1.** Summary of Ageing Well Characteristics Across Discussed Models

Models	Characteristics of ageing well													
	Avoidance of disease /disability	Good physical functioning	Good cognitive functioning	Engagement in social relationships	Engagement in society/productive activity	Spirituality	Adaptation	Resilience	Purpose/meaning in life	Autonomy	Self-acceptance	Environmental mastery	Personal growth	Expertise in at least one area
Rowe and Khan’s Model of Successful Ageing (1987, 1997)	✔	✔	✔	✔	✔									
Crowther et al.’s (2002) Inclusion of Spirituality	✔	✔	✔	✔	✔	✔								
Ryff’s Model of Psychological Wellbeing (1989)				✔							✔	✔	✔	
Baltes and Baltes (1990) Model of Selective Optimisation with Compensation							✔							
Schulz and Heckhausen’s (1996) Lifespan Model of Successful Ageing		✔	✔	✔				✔						✔
Reker’s (2009) Successful Ageing Scale	✔	✔	✔	✔	✔		✔	✔		✔	✔			
Pruchno et al.’s (2015) Successful Ageing as Resilience								✔						

### *Successful Ageing as Resilience* (Pruchno et al., 2015)

In contrast to theories where successful ageing is determined by meeting specific criteria, Pruchno et al. (2015) proposed that the ability to age successfully is a product of resilience in response to experiencing hardship across the lifespan. Resilience is the ability to adapt to, and cope with, adversity (Masten & Reed, 2001). Masten and Reed (2001) argued that resilience is not an innate trait that people either do or do not possess but is a characteristic that can be fostered and developed. As such, and according to Pruchno et al. (2015), individuals can age successfully by developing resilience. The authors note that everyone experiences some form(s) of adversity throughout life. Older people, by virtue of having lived longer, have experienced more losses (e.g., social, role, functioning). These losses could be considered adversities. This conceptualization of successful ageing could be seen as more inclusive than previous models as those individuals with health conditions and disabilities can age successfully. The avoidance of disease and disability is a feature of some models of successful ageing, and consequently, those individuals with a disability are precluded from ageing successfully (Martinson & Berridge, 2015).

### The Inclusion of Spirituality

Although endorsing the components of Rowe and Kahn’s (1997) model of successful ageing, some authors have proposed expanding the model to include positive spirituality (Crowther et al., 2002; Flood, 2005). Positive spirituality is defined as the aspects of religion or spirituality that align with enhancing life and increasing relationships with (a) the self, (b) other people, and (c) a “transcendent force” (e.g., God; Crowther et al., 2002). These authors assert that positive spirituality can assist individuals to find, or enhance, meaning and purpose, can help to restore feelings of control and support, and can increase social connection with others (e.g., attending church). In support of the position that spirituality should be included in models of ageing successfully, Zanjari et al. (2016) found spirituality was the fourth most prevalent theme mentioned in relation to perceptions of ageing successfully; themes mentioned by a greater proportion of the cohort were social well-being, psychological well-being, and physical health.

## Limitations of Successful Ageing

### Definitions and Measurements

Although successful ageing originally comprised, in part, being physically healthy (see Rowe & Kahn, 1987), later conceptualizations focus on psychological wellness (e.g., Ryff, 1989), adapting to age-related changes (e.g., Baltes & Baltes, 1990), and the importance of a healthy lifestyle (e.g., Reker, 2009). This more inclusive approach addresses the outdated assumption that being physically or cognitively less capable is equivalent to ageing poorly. Steverink (2014) described how an individual with reduced physical, cognitive, or social function may rate their well-being as high, whereas an individual who is considered high functioning in these areas may be low in subjective well-being. Consequently, Steverink (2014) asserted that objective measures of function are not the most appropriate indicators of successful ageing and, instead, subjective measures of well-being would better represent success. Relatedly, inconsistencies in definitions make it difficult to measure successful ageing. The lack of agreement creates ambiguity in determining whether heterogeneity of successful ageing is present within the ageing population, or if research outcomes are a product of different definitions and, consequently, measurement tools.

Since the term “successful ageing” was coined some 40 years ago, there have been significant societal shifts that have shaped people’s ability to age successfully. Examples include the legalization of homosexuality and same-sex marriage in many United Nations countries (Pew Research Center, 2023; Statista, 2023), the election of women to leadership positions (UN Women, 2023), and legal access to birth control (Liao & Dollin, 2012). These shifts meant that new roles, experiences, and opportunities were available to members of certain groups which, in turn, influenced (a) their ability to age successfully, and (b) what is considered important in ageing successfully. By way of example, access to contraception, for birth control or menstrual regulation, can (both directly and indirectly) improve physical and mental health (e.g., Herd et al., 2016; Karlsson et al., 2021; Robertson et al., 2021; Zorbas et al., 2015). According to fundamental theories (Crowther et al., 2002; Rowe & Kahn, 1997; Schulz & Heckhausen, 1996), improved physical health likely contributes to successful ageing. Furthermore, the ability to make decisions and advocate for oneself (e.g., choosing to use contraceptives or get married) aligns with autonomy, which Ryff (1989) deemed a defining characteristic of successful ageing. There are several ways societal progress enhances the ability of individuals to age successfully. Sensitivity to diversity, identity, and social disparities deserve consideration when conceptualizing successful ageing and aging well, and as society advances our perspective of factors that contribute to successful ageing require reconsideration.

### Dichotomy of Success

The dichotomy of successful versus unsuccessful ageing has been a contentious point (see Martinson & Berridge, 2015), with some authors asserting it is a form of ageism (Angus & Reeve, 2006). The use of “successful” or “successfully” implies a categorical all-or-nothing approach to ageing. That is, one is either ageing successfully or they are not. Holstein and Minkler (2003) explained that failing to attain what is deemed “success” can be considered failure, and this can reinforce negative stereotypes (e.g., older bodies are physically limited), fear around ageing, and ageism. Martinson and Berridge (2015) highlighted concerns regarding “successful ageing” being an exclusionary concept. Their review emphasized the culturally biased and, for many, ill-fitting criteria used as markers of successful ageing. Given this finding, and although Depp and Jeste (2006) argued that demographic variables are not associated with one’s ability to age successfully, a fundamental facet of appropriately researching successful ageing is understanding the influence of socioeconomic factors, gender, and race (Holstein & Minkler, 2003).

To explain, people’s privilege and circumstances affect the choices available to, and made by, them throughout life. Access to nutritious food is an example of an external factor that could influence one’s ability to meet existing criteria for ageing successfully (Holstein & Minkler, 2003). With the exception of Pruchno et al.’s (2015) view of successful ageing being a pattern of resilience, other models rely on health and functioning, connection and engagement, and autonomy. As such, disregarding existing inequalities beyond one’s control can be harmful in that marginalized groups may not be able to meet the criteria of ageing “successfully.” Future research into ageing should be conducted with an intersectional lens, thus considering the various facets of identity in the context of society, privilege, and oppression.

To reiterate, the term “successful ageing” is problematic due to the implied dichotomy of successful versus unsuccessful ageing and, accordingly, precludes many from reaching “success” (e.g., those with disabilities). As such, “ageing well” is proposed as an alternate term. Although “ageing well” is not new (see George & Clipp, 1991; Wahl et al., 2012), it will be argued that this term, once properly defined, would better capture current understandings of optimal experience of late adulthood. Ageing well has been used by several authors (e.g., Angus & Reeve, 2006; Chapman, 2005; Williamson, 2005), which avoids the difficulty of the potentially exclusionary, dichotomous conceptualization associated with “successful ageing.” However, the literature that has used this term does not provide a clear consensus definition of “ageing well” any more than does the successful ageing literature. The list of factors to be considered as defining or contributing to ageing well has changed over time (Chapman, 2005; Menassa et al., 2023; Quigley et al., 2022). What we offer is a reflection of the contemporary agenda for ageing well that emerges from both past theory and recent practice.

To address other identified shortcomings, we propose that future research investigating ageing well considers the influence of factors that can fall outside of one’s control on the ability to age well. These include, but are not limited to, socioeconomic factors, gender, race, and exposure to negative stereotypes. Some researchers (e.g., Wahl et al., 2012) note that it is important not to decontextualize individuals when investigating ageing well. That is, they should not be considered distinct from the environment in which they age. This aligns with Schulz and Heckhausen’s (1996) assertion that any appraisal of success in ageing ought to be tempered by the individual’s genetics and sociocultural opportunities, given the potential for these factors to affect accessibility to “success.” Given these issues, it seems important that researchers identify a set of factors that facilitate ageing well (e.g., social connectedness, adaption, purpose in life); these can each be measured, but one would not need to have all of them to age well, nor would a total score on a measure assessing a combination of these factors provide an ageing well score. Rather, the presence of such factors might explain why an individual is ageing well, and their absence might identify why an individual is not thriving in later life.

## Ageing Well in Practice

A lack of an agreed-upon definition of successful ageing, or ageing well, in research has real-world implications. This can be seen in the diverse approaches being taken in different jurisdictions to frame government policy on ageing. Worldwide, many jurisdictions are moving to models of ageing well that include psychological factors, mirroring the transition in the literature. For example, European policies are moving away from successful ageing in favor of “active ageing” to provide a more holistic approach (Foster & Walker, 2015). Foster and Walker (2015) argued that “active aging” comprises, for instance, engagement in meaningful activities that contribute to the individual’s well-being, preventative health intervention, intergenerational solidarity, and respecting the population’s diversity. An example from the United States is that of Texas where, consistent with Rowe and Kahn’s (1997) view, older Texans identified physical health as a priority in ageing well. They also identified sociopsychological factors that are important to ageing well, including access to social and recreation opportunities, and access to community-based services and support (Texas Health and Human Services, 2023). Within the United Kingdom, “Ageing Well in Wales” focused on (a) age-friendly communities, (b) prevention of falls, (c) dementia-supportive communities, (d) providing learning and employment opportunities, and (e) addressing loneliness and isolation (Older People’s Commissioner for Wales, 2016). They noted these as key areas to help support independence, participation, and health (see Rowe & Kahn’s [1997] model), well-being (see Ryff’s [1989] model), and the economy. As can be seen, approaches to ageing well differ across countries and even jurisdictions within countries.

The lack of an agreed-upon definition of ageing well is clearly illustrated in government policy in Australia. As the Australian Federal Government has not provided a formal strategy or policy regarding ageing well, each state has interpreted and responded to ageing well in different ways. Of interest, states appear to have adopted facets of the models described earlier during policy development. The Government of South Australia’s ageing well plan (Department for Health and Wellbeing, 2020), for example, was based on a study of 1,211 older adults by the Australian Centre for Social Innovation (2018). Three themes of importance in relation to ageing well emerged, including (1) being able to manage change (e.g., supports for older adults to assist with grief and loss; adaptation is a feature of Baltes and Baltes’ [1990] SOC model), (2) having meaningful connections (e.g., genuine opportunities for social connection and activities; see Rowe & Kahn, 1997; Ryff, 1989; Schulz & Heckhausen, 1996), and (3) the importance of home (e.g., older adults reported that their home provides a sense of belonging and autonomy, and many wish to remain living at home; autonomy is a feature of Ryff’s [1989] model). Financial well-being and dismantling ageism were also considered important in supporting older Australians to age well (The Australian Centre for Social Innovation, 2018).

In addition to these themes, other Australian states (i.e., Victoria, New South Wales) have identified additional features or focus areas to support ageing well (Commissioner for Senior Victorians, 2020; Department of Communities and Justice, 2021) including, but not limited to, having a positive attitude, having a purposeful and meaningful life, mutual respect, keeping up with the changing world, and ensuring older people (a) can participate in inclusive communities (e.g., making workplaces age-friendly) and (b) are informed and resilient (e.g., engaging with advocacy groups to develop long-term policies). The notion of keeping up with the changing world aligns with adaptability (Baltes & Baltes, 1990), whereas purpose in life is a key factor in Ryff’s (1989) model. Furthermore, social and life engagement are components of several successful ageing theories (see Table 1). Furthermore, some of these focus areas support existing theories of ageing successfully, such as autonomy (e.g., giving older Australians the resources to make decisions about their health and engagement; see Ryff’s [1989] model), adaptation and personal growth (e.g., providing digital literacy courses; see Baltes and Baltes’ [1990] SOC model and Ryff’s [1989] model, respectively), and resilience (e.g., providing culturally appropriate information to support change navigation; see Pruchno et al., 2015; Schulz & Heckhausen, 1996).

Although research in the Australian context identifies factors consistent with international interpretations of ageing well, what is new here is that older adults recognized a reduction in ageism as an important and consistent theme, which is not part of fundamental models of ageing well. That said, the literature has acknowledged that the ability to age well could be improved by reducing negative stereotypes associated with ageing (Bryant et al., 2012). Ageism has a negative association with well-being among older people (Kang & Kim, 2022), and the adoption of age-related stereotypes by older people can negatively influence their self-perceptions (Rothermund & Brandtstädter, 2003). This, in turn, can affect longevity (i.e., those with greater negative self-perceptions of ageing live shorter lives; Levy et al., 2002) and reduce cognitive performance (Lamont et al., 2015). Together, these findings suggest that models of ageing well should consider the impact of exposure to negative age-related stereotypes and/or an individual’s adoption of age-related stereotypes.

## Conclusion

In summary, varied definitions and models of successful ageing and ageing well exist, and although there remains no universally accepted definition, common themes have been identified (see Table 1). The lack of agreement regarding the definition is problematic and has implications for research, policy development, and some individuals’ ability to age “successfully” (e.g., those with disabilities). Although several key conceptualizations of successful ageing have been reviewed here, each with its own merits, current conceptualizations do not adequately capture the factors contributing to successful ageing, particularly in response to inclusivity, diversity, and societal changes since the term was first coined. The measure provided by Reker (2009) attempted to address this by amalgamating several fundamental theories, resulting in the most well-developed of the conceptualizations discussed, and allows for a noncategorical approach (i.e., people can fall on a continuum between success and lack thereof). However, given this model is still flawed (e.g., the inclusion of absence of disease/disability), an opportunity exists for the re-conceptualization of this construct. As such, “ageing well” is proposed as an alternate term. Ageing well provides an opportunity to address other important limitations associated with ageing “successfully.” These limitations include a failure to take an individual’s context into consideration. Wahl et al. (2012) proposed that assessing the person–environment interaction may facilitate this objective. The point here is that individuals should not be precluded from “ageing well” based on circumstances beyond their control. Important factors that could contribute to a definition of ageing well have been provided (e.g., resilience, social relationships), and an appropriate definition of ageing well now needs to be developed and validated by gathering empirical data from older adults. Here, a participatory approach that aims to have older adults elaborate on what makes life better or worse in later years will likely be important in developing an accurate definition of ageing well. It is worth noting that, without a clear understanding of what contributes to ageing well, government policies might not actually help people age well.

## Data Availability

This article does not report data and therefore the preregistration and data availability requirements are not applicable.
